# Cerebrospinal Pharmacokinetic Modeling and Pharmacodynamic Simulation of High-Dose Cefazolin for Meningitis Caused by Methicillin-Susceptible *Staphylococcus aureus*

**DOI:** 10.3390/antibiotics14101008

**Published:** 2025-10-11

**Authors:** Tetsushu Onita, Kazuro Ikawa, Noriyuki Ishihara, Hiroki Tamaki, Takahisa Yano

**Affiliations:** 1 Department of Pharmacy, Shimane University Hospital, 89-1 Enya-cho, Izumo 693-8501, Japan; 2 Department of Clinical Pharmacotherapy, Hiroshima University, 1-2-3 Kasumi, Minami-ku, Hiroshima 734-8551, Japan

**Keywords:** cefazolin, meningitis, staphylococcal, renal

## Abstract

**Background:** Cefazolin is being increasingly used to treat central nervous system infections caused by methicillin-susceptible *Staphylococcus aureus* (MSSA) to mitigate the side effects of existing anti-Staphylococcal drugs. This study aims to develop a cerebrospinal pharmacokinetic (PK) model to predict the cefazolin concentration in cerebrospinal fluid (CSF) and to individualize the dosing regimen for MSSA meningitis. **Methods:** A cerebrospinal PK model was developed based on the existing literature and used to estimate the probability of attaining PK/ pharmacodynamic (PD) targets. These targets were set as 100% time above the minimum inhibitory concentration (T > MIC) in CSF. The cerebrospinal PK/PD breakpoint was defined as the highest MIC at which target attainment probability in CSF was ≥90%. The mean CSF/serum ratio estimated from the literature was 0.0525 after a dose of 1–3 g (sampling time: 1–9 h after dose) in adult patients with suspected meningitis. This ratio was incorporated into this PK model based on a hybrid approach. **Results:** For patients with creatinine clearance (CL_cr_) = 90 mL/min, the cerebrospinal PK/PD breakpoint MICs of continuous infusion regimens (6–12 g/day) reached 0.5 µg/mL, which can inhibit the growth of 90% of the MSSA population (MIC_90_). Furthermore, for patients with renal dysfunction (CL_cr_ = 30 mL/min), a dose reduction (4 g/day) may be required to avoid excessive drug exposure. **Conclusions:** High-dose continuous infusion of cefazolin may be appropriate for MSSA meningitis in patients with normal renal function, while dose adjustments are needed for those with renal impairment.

## 1. Introduction

Central nervous system (CNS) infections caused by bacteria are life-threatening conditions with a high mortality rate worldwide. Methicillin-susceptible *Staphylococcus aureus* (MSSA) is one of the most common bacterial pathogens causing CNS infections such as shunt-related meningitis [1]. Nafcillin and oxacillin are anti-staphylococcal penicillin-like antibiotics that are used as the first-line treatment for MSSA-mediated shunt-related meningitis [1,2,3]. However, these antibiotics are associated with toxicity (e.g., fluid retention, elevated liver enzymes, interstitial nephritis) and are unavailable in some countries, including Japan [4]. Cefazolin, a first-generation cephalosporin antibiotic, has excellent activity against MSSA and has frequently been used to treat MSSA infections [5,6,7,8,9]. Still, it is not recommended for treating CNS infections due to its low penetration into the CNS [10]. However, several recent reports have suggested that high-dose regimens of cefazolin are effective against MSSA meningitis [11,12,13,14], suggesting that the use of cefazolin for CNS infections should be reconsidered.

Pharmacokinetic (PK)/pharmacodynamic (PD) theory has previously been used to optimize antimicrobial treatment [15]. The antimicrobial activity of β-lactam antibiotics such as cefazolin is generally dependent on the exposure time during which the drug concentrations remain above the minimum inhibitory concentration for the bacteria (T > MIC) [16,17]. Although there are reports that simply measured the cefazolin concentrations in CSF [11,12], none of the previous reports has described cerebrospinal PK or assessed site-specific PK/PD with mathematical modeling and stochastic simulation. Furthermore, the previously reported data [11,12,13] on cefazolin CSF concentrations were observed in patients with normal renal function, and there are no data for patients with impaired renal function. Therefore, a mathematical model for predicting CSF concentrations can be valuable. To verify the appropriateness of high-dose regimens, a stochastic PK/PD evaluation is required from a quantitative perspective. Therefore, this study aims to develop a cerebrospinal PK model that predicts the cefazolin concentration in CSF using existing data, and to optimize the dosing regimen considering the sensitivity of MSSA to cefazolin and patient backgrounds.

## 2. Results

### 2.1. Model Validation

**Figure 1:**
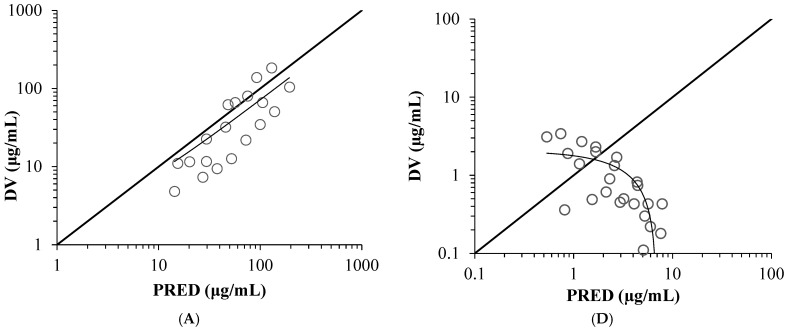

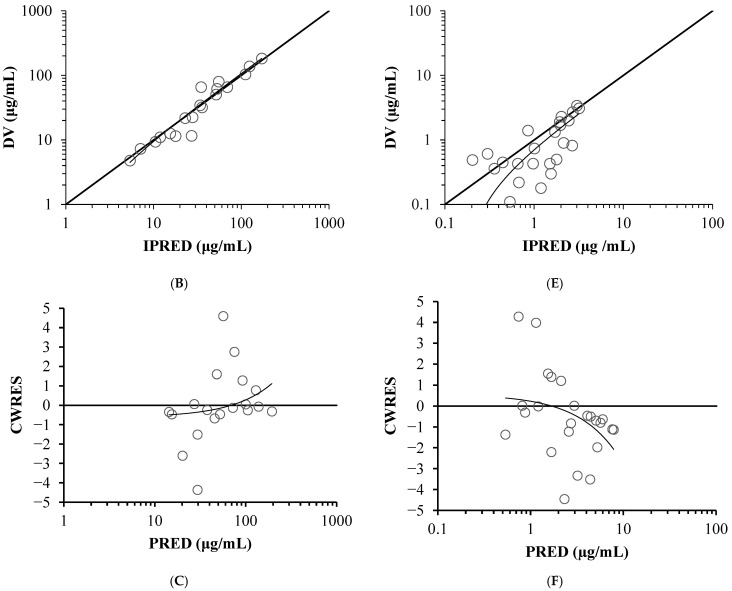
Scatter plots of the final population PK model of cefazolin. Observed values (DV) versus population-predicted values (PRED) (**A**) or individual-predicted values (IPRED) (**B**) in plasma, conditional weighted residual (CWRES) versus population-predicted values in plasma (**C**), observed concentrations versus population-predicted values (PRED) (**D**) or individual-predicted values (IPRED) (**E**) in cerebrospinal fluid, and CWRES versus population-predicted values in cerebrospinal fluid (**F**).

All final model parameters (ηKP_CSF_, ηQ_CSF_ and ηV_CSF_) were estimated to be within the 95% confidence intervals based on sampling importance resampling algorithm. Figure 1 shows the goodness-of-fit plots of the plasma and cerebrospinal fluid (CSF) concentrations. Scatter plots of the observed concentration (DV) vs. population-predicted concentration (PRED) or individual-predicted values (IPRED), and conditional weighted residuals (CWRES) vs. PRED in plasma and CSF indicated some bias.

**Figure 2:**
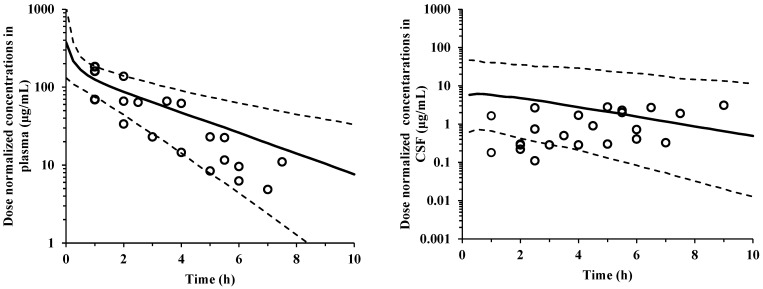
Dose-normalized visual predictive check plots representing observed plasma and cerebrospinal fluid (CSF) concentrations (open circles) normalized to 2 g of cefazolin. For each graph, the dotted line represents the predicted 95% confidence interval (from 2.5th to 97.5th percentiles) and the solid line represents the 50th percentile.

Furthermore, dose-normalized visual predictive checks were performed for the observed and predicted concentrations in plasma and CSF vs. the time curves of cefazolin (Figure 2). Most of the observed values were within the predicted 95% confidence intervals for the 2.5th, 50th, and 97.5th percentile points.

### 2.2. PK/PD Evaluation

**Figure 3:**
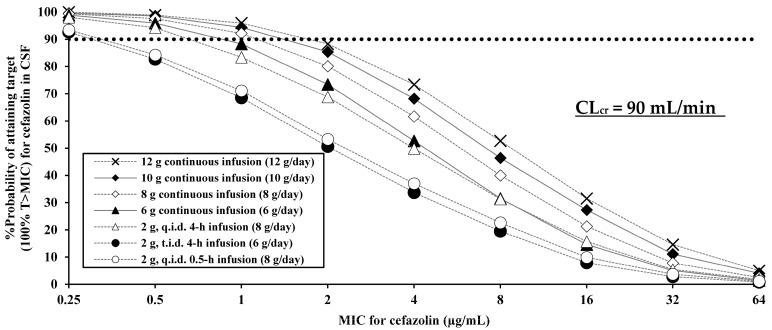

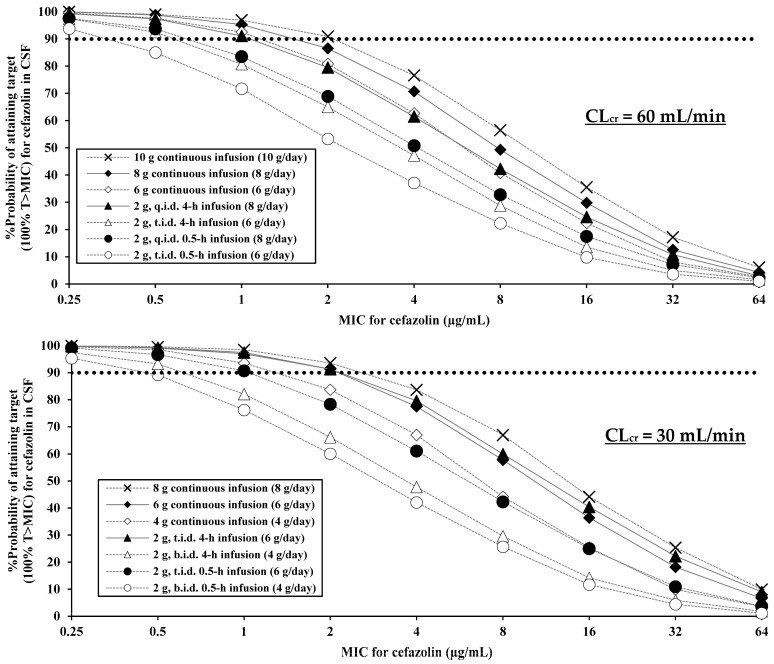
Probabilities of attaining pharmacokinetic/pharmacodynamic target (100% T > MIC) in cerebrospinal fluid for cefazolin at specific MICs using twice-daily (b.i.d.), three-times-daily (t.i.d.), four-times-daily (q.i.d.) regimens and continuous infusions. The dotted lines represent 90% probability. CL_cr_; Creatinine clearance, T > MIC; time above minimum inhibitory concentration.

We estimated the probabilities of target attainment in the CSF based on the final model using different dosing regimens (Figure 3). For 100% time above the minimum inhibitory concentration (T > MIC), the cerebrospinal pharmacokinetic/pharmacodynamic (PK/PD) breakpoints were defined as the highest MIC at which the target attainment probability in CSF was >90% (Table 1).

For typical patients with creatinine clearance (CL_cr_) = 90 mL/min, the cerebrospinal PK/PD breakpoint MICs were as follows: 0.25 μg/mL for 2 g four times daily (0.5 h infusion) and 2 g three times daily (4 h infusion); 0.5 μg/mL for 2 g four times daily (4 h infusion) and 6 g continuous infusion; 1 μg/mL for 8 g, 10 g, and 12 g, each as a continuous infusion.

For typical patients with CL_cr_ = 60 mL/min, the cerebrospinal PK/PD breakpoint MICs were as follows: 0.25 μg/mL for 2 g three times daily (0.5 h infusion); 0.5 μg/mL for 2 g four times daily (0.5 h infusion) and 2 g three times daily (4 h infusion); 1 μg/mL for 2 g four times daily (4 h infusion), 6 g and 8 g as continuous infusions; 2 μg/mL for 10 g continuous infusion.

For typical patients with CL_cr_ = 30 mL/min, the cerebrospinal PK/PD breakpoint MICs were as follows: 0.25 μg/mL for 2 g twice daily (0.5 h infusion); 0.5 μg/mL for 2 g twice daily (4 h infusion); 1 μg/mL for 2 g three times daily (0.5 h infusion) and 4 g continuous infusion; 2 μg/mL for 2 g three times daily (4 h infusion), 6 g and 8 g as continuous infusions.

## 3. Discussion

The efficacy and feasibility of high-dose regimens for MSSA associated meningitis have not been evaluated from a quantitative perspective or optimized based on the patient’s renal function. In this study, a cerebrospinal PK model of cefazolin was developed, integrating existing data. The PD for high-dose regimens of cefazolin in patients with MSSA meningitis was also evaluated, considering renal function.

In the cerebrospinal PK model, KP_CSF_ was calculated based on the observed serum and CSF concentrations, as described by Ikuno H et al. [18]. The mean CSF/serum ratio estimated from their report was 0.0525 after a 1–3 g dose (sampling time: 1–9 h after dose) in adult patients with suspected meningitis. A previous review article of Antosz K. et al. reported a CSF/plasma concentration ratio ranging from 0.03 to 0.12 (inflamed) [14]. The current results are within the previous report and reasonable. The previously reported data used to calculate KP_CSF_ in this study included two and six patients with CSF protein levels over 300 mg/dL and under 100 mg/dL, respectively. The CSF/serum concentration ratio in patients with CSF protein levels over 300 mg/dL tended to be higher than in those with CSF protein levels under 100 mg/dL. However, quantitative incorporation of CSF protein levels into KP_CSF_ as a covariate was difficult due to the small sample size. In cases where inflammation has decreased since the start of treatment, higher doses may be required.

A CSF PK model for cefazolin was generated using hybrid modeling by leveraging previously reported PK parameters and incorporating physiological parameters. Since the specific CSF flow clearance of a drug depends mainly on its physiological factor, the system-to-CSF and CSF-to-system clearance values were supposed to be the same, and hence were set as the CSF flow in the hybrid modeling. All final model parameters (ηKP_CSF_, ηQ_CSF_ and ηV_CSF_) were estimated to be within the 95% confidence intervals based on the sampling importance resampling algorithm. For the goodness-of-fit plots (Figure 1), the trend line indicated some bias which raised concerns about the validity of the model. However, we comprehensively judged that the goodness-of-fit plots distribution were generally uniform across the diagonal line, that most of the observed values in the dose-normalized visual predictive check results (Figure 2) were within the 95% confidence interval. Furthermore, the uncertainty in model validation may be due to the small number of CSF samples. These are considered as a limitation of this study.

Regarding the cerebrospinal PD evaluation, the PK/PD breakpoints (100% T > MIC) in CSF of dosing regimens with continuous infusion in typical patients with CL_cr_ = 90 mL/min reached 0.5 µg/mL for MIC_90_ = MSSA (Table 1). Meanwhile, since these breakpoints were lower for 0.5 h infusion regimens in typical patients (maximum 0.25 μg/mL), these regimens are not recommended. The PD simulation results demonstrated the appropriateness of high-dose administration, especially in cases with good renal function. As for the appropriateness of PD simulation, Grégoire M et al. reported that the mean total cefazolin concentrations in CSF after continuous infusion of 8 g daily in a case report with normal renal function (glomerular filtration rate: 60 to 106 mL/min per 1.73 m^2^ after six weeks) was 6.1 µg/mL [11]. Le Turnier P et al. reported that median CSF concentrations after mainly continuous infusions of 8 g daily in subjects with normal renal function (the median glomerular filtration: 136 mL/min) was 2.8 µg/mL (ranging from 2.1 to 5.2 µg/mL) [12]. The median concentration of our simulation for continuous infusion of 8 g daily in typical patients with CL_cr_ = 90 mL/min was 5.7 µg/mL (95% confidence intervals: 0.5 to 62.4 µg/mL) and covered the values of these reports. Furthermore, Novak AR et al. reported that the median trough CSF concentration for 2 g three times daily (6 g/day) in subjects with normal renal function (median CL_cr_: 115 mL/min) was 1.59 µg/mL (ranging from 0.7 to 2.2 µg/mL) [13]. The median concentration of our simulation for the same dosing regimen in patients with CL_cr_ = 90 mL/min was 1.2 µg/mL (95% confidence intervals: 0.05–25.1 µg/mL), which was similar to the previous values. (Supplementary Table S1) Furthermore, Pitcock C et al. reported that the probability attaining of 100% T > MIC in CSF was more than 90% for 6–12 g/day continuous infusion of cefazolin at 0.5 µg/mL in patients with mean creatinine clearance of 115 mL/min [19]. Comparison with these reported values indicates that our PD simulations are generally reasonable. Therefore, concerns about our model could be resolved with comprehensive judgment from a viewpoint of this external PD validation. Notably, all the values reported above [11,12,13] were from patients with good renal function, and the recommendation of high-dose regimens is limited to them, because high exposure to cefazolin, especially in patients with impaired renal function, has been reported to cause seizures [20]. In fact, the safety simulation to avoid cefazolin-related neurotoxicity showed that even in patients with impaired renal function, dose reduction could reduce the probability of exceeding the neurotoxicity related concentration (64 µg/mL) to less than 10% (Supplementary Table S2). Because a target value for toxicity (e.g., neurotoxicity, nephrotoxicity, or seizures) was not established, the median of cefazolin CSF concentrations (64 µg/mL) in three cases with generalized seizures [20] was used as a reference concentration for safety simulation of cefazolin regimens. Therefore, it is important to adjust, in order to avoid excessive drug exposure, the dose based on the renal function of the patient by referring to the PK/PD breakpoints.

This study mainly has four limitations. First, the population included patients with suspected, and not confirmed meningitis, and hence, the CSF/serum concentration ratio might be underestimated for patients diagnosed with bacterial meningitis. However, since CSF sample collection is challenging and the number of individual cefazolin CSF concentrations previously reported was very low, the inclusion of these populations was considered acceptable. Second, this cerebrospinal model of cefazolin was developed using a PK model in blood and CSF concentration data in an adult population. Therefore, the model cannot be applied to a pediatric population. Third, there are no reports demonstrating the clinical efficacy of cefazolin for meningitis in patients with renal dysfunction, and the appropriateness of dosing regimens proposed in this study has not yet been clinically verified. Therefore, clinical confirmation of the proposed regimens is necessary in patients with impaired renal function. Fourth, this study did not consider the protein binding in CSF due to lack of the detailed data. However, the free cefazolin concentration in CSF (*n* = 5), the total and free concentrations were similar [11], which suggested dosing regimens in this study may be appropriate.

## 4. Materials and Methods

### 4.1. Cerebrospinal PK Modeling

**Figure 4:**
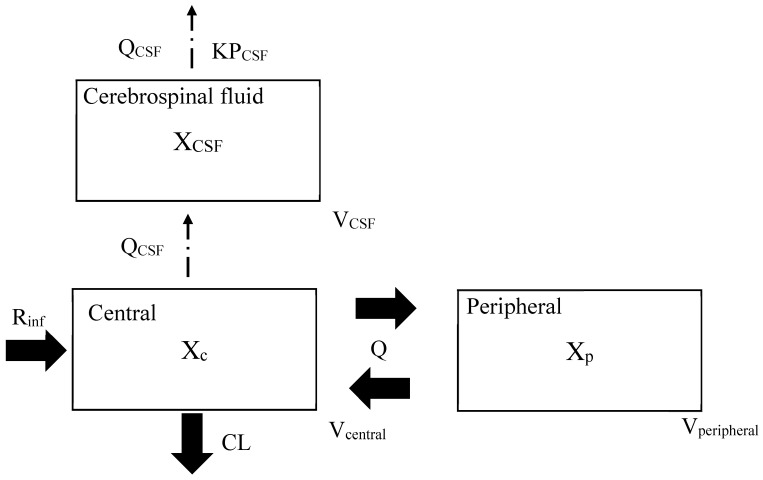
Cerebrospinal pharmacokinetic model of cefazolin X_c_, X_p_ and X_CSF_ are the amount of cefazolin (mg) in the central, peripheral and cerebrospinal fluid (CSF) compartments; R_inf_, the drug infusion rate (mg/h); CL, clearances (L/h); V_central_, V_pheripheral_ and V_CSF_, distribution volumes of the central, peripheral and cerebrospinal fluid compartments (L); Q, central–peripheral intercompartmental clearance (L/h); Q_CSF_, CSF flow (L/h); KP_CSF_, CSF to plasma partition coefficient.

The cerebrospinal pharmacokinetics of cefazolin were described using the following hybrid model (Figure 4), which includes several physiological parameters, such as body fluid flow and volume. This model is partially connected to the conventional PK model and was therefore utilized for the site-specific PK/PD analysis [21,22,23,24].

In the modeling, previously reported PK parameters were leveraged and physiological parameters were incorporated because the observed concentration data in both CSF and plasma were very limited and too small for full modeling. Thus, PK models predicting blood concentrations were systematically searched using the keywords “cefazolin” and “population pharmacokinetics”. The PK model used in the hybrid model to predict plasma concentrations should be simpler (not dealing with protein-unbound concentrations or concentrations in tissue or fluid) and should not be for special populations. Therefore, the following exclusion criteria were set: (1) in animals or combined with other drugs; (2) population PK analysis methods other than nonlinear mixed effects modeling; (3) special populations (pediatric, pregnant women, dialysis, etc.); (4) predicting drug concentrations in tissues and body fluids, and (5) predicting free drug concentrations. Through this defined process, the PK model reported by Lanoiselée J. et al. [25] was selected as the model predicting plasma concentration. As the next step, reported Q_CSF_ (CSF flow in L/h) and V_CSF_ (CSF volume in L), as physiological parameters [26], and KP_CSF_ (CSF-to-plasma partition coefficient) estimated from a previous report [18], were connected to the PK model [25]. Thus, the hybrid model is composed of three compartments. The CSF PK model was based on the following equation.

dX(central)/dt = R_inf_ − (CL/V_central_ + Q/V_central_)∗X(central) + Q∗X(peripheral)/V_peripheral_

dX(peripheral)/dt = Q∗X(central)/V_central_ − Q∗X(peripheral)/V_peripheral_

dX(CSF)/dt = Q_CSF_∗X(central)/V_central_ − Q_CSF_∗X(CSF)/V_CSF_/KP_CSF_

In the formulas, X(central), X(peripheral), and X(CSF) are the amounts of the drug (mg) in the central, peripheral, and CSF compartments, respectively; R_inf_ is the rate of infusion (mg/h); CL is the clearance (L/h) from the central compartment; V_central_ and V_peripheral_ are the volumes of distribution (L) of the central and peripheral compartments, respectively; and Q is the central–peripheral intercompartmental clearance (L/h). The model parameters are listed in Table 2. In the blood compartments, the fixed-effects parameters (θCL, θV_central_, θQ, and θV_peripheral_) and the interindividual variability (ηCL, ηV_central_, ηQ, and ηV_peripheral_) were obtained from cefazolin population PK parameters [25]. The fixed-effects parameters were ascertained as follows: CL = 2.86 (L/h) (CL_cr_, which was calculated by the CKD-EPI formula, was incorporated as a covariate), V_central_ = 5.2 (L), Q = 10.9 (L/h), and V_peripheral_ = 4.56 (L). Q_CSF_ (CSF flow in L/h) and V_CSF_ (CSF volume in L) were used as physiological fixed-effects parameters, were fixed as follows: Q_CSF_ = 0.021 (L/h), and V_CSF_ = 0.092 (L) [26]. The CSF/serum concentration ratio at the same sampling points of serum and CSF estimated from literature values incorporated into KP_CSF_ (CSF-to-plasma partition coefficient) [18]. The demographic information from the literature data quoted in this study is represented in the Supplementary Materials (Supplementary Table S3). As bioanalytical methods for the measurement, a liquid chromatography system was used for blood concentrations in the PK parameters [25] and a bioassay was used for CSF concentrations [18]. The structure of the PK model for predicting blood concentration was a two-compartment model. For covariate analysis of this PK model, age, total body weight, body mass index, lean body weight, and CL_cr_ (mL/min) according to the Cockcroft-Gault formula and the Chronic Kidney Disease-Epidemiology Collaboration (CKD-EPI) formula were tested with stepwise procedure, CL_cr_ according to CKD-EPI formula was incorporated as the only covariate for CL in the final model [25]. Additionally, to improve the robustness of the final model, we performed a $PRIOR subroutine analysis [27]. Using $PRIOR NWPRI, we assigned prior information to $THETAP by referencing the RSEs of previous studies [18,25,26] used to develop the model. As a result, the parameters (ηKP_CSF_, ηQ_CSF_, ηV_CSF_) analyzed with prior were similar to those without prior, and the RSEs of KP_CSF_ were reduced from 203% to 18%. The results of the sampling importance resampling algorithm showed that estimates were within the 95% CI for the model without priors, but were outside the range for the model with priors. Therefore, referring to Marshall et al.’s approach [28] and the results of the sampling importance resampling algorithm, this type of hybrid model building without priors was adopted (Supplementary Figure S1). The PK model predicting CSF concentrations was developed using the NONMEM (version 7.4; ICON Public Limited Company, Dublin, Ireland).

### 4.2. Estimation of CSF/Serum Concentration Ratio

Cefazolin concentration data for paired serum and CSF were obtained from 8 adult patients with suspected meningitis, of which 19 serum samples were from 7 patients and 24 CSF samples were from 8 patients [18]. To assess the penetration into the CSF, the CSF/serum concentration ratio was estimated from the serum and CSF at the same sampling points and the times after administration were investigated from the literature data. Mean CSF/serum concentration ratio at the same sampling point was 0.0525 (Standard error: 0.0548) after a 1–3 g dose (sampling time: 1–9 h after dose). Regarding the CSF findings, the CSF protein levels exceeded 300 mg/dL (with inflammation) in two patients, and the mean CSF/serum concentration ratio was 0.081, while six patients had levels below 100 mg/dL (without inflammation), with a mean CSF/serum concentration ratio of 0.038. Regarding to the comparison between sexes, the mean CSF/serum concentration ratio at the same sampling points for males (*n* = 4) and females (*n* = 4) was 0.0611 and 0.0457, respectively, and there were no significant differences.

### 4.3. Model Validation

The robustness of the parameters was tested using a sampling importance resampling algorithm applicable when the analysis population is small with the Perl-speaks-NONMEM software 5.4.0 [29]. The 95% confidence intervals of the parameters from this algorithm (sampling: *n* = 1000 and resampling *n* = 1000) were compared with the estimates of the final population model. The final model was validated using goodness-of-fit plots, which included observations versus predictions, CWRES versus prediction, and dose-normalized visual predictive checks. The 95% prediction interval of the drug concentration time course was obtained using the values from the 2.5% point to the 97.5% point of concentrations at each time point by performing 1000 simulations with the final parameter estimates.

### 4.4. PK/PD Simulation

We randomly generated 1000 cases of the seven fixed-effects parameters θ_i_ (CL, V_central_, Q, V_peripheral_, KP_CSF_, Q_CSF_, V_CSF_) using the $SIMULATION command in NONMEM according to each mean estimate and the interindividual variance in the final model. Model equations were generated using these seven θ_i_ values to estimate the CSF concentrations of cefazolin at steady state for different dosing regimens (0.5 h, 4 h and continuous infusion). The time point at which the CSF concentration coincided with a specific MIC value (0.25–64 μg/mL) was determined, and the drug exposure time above the MIC (T > MIC) was calculated as the cumulative percentage during 24 h for different dosing regimens. The probability of target attainment (%) at a specific MIC was defined as the proportion that achieved 100% T > MIC as the PK/PD target. The total CSF concentration was not adjusted for the free fraction because the protein binding of cefazolin in CSF is currently unknown. Antimicrobial susceptibility of MSSA was derived using surveillance results in 2018 from the Four Academic Societies Joint Antimicrobial Susceptibility Surveillance Program (MIC_50_ = 0.5 µg/mL, MIC_90_ = 0.5 µg/mL) [30].

## 5. Conclusions

In this study, a cerebrospinal PK model of cefazolin was developed. The modeling and simulation analyses suggested that high-dose continuous infusion regimens (6–12 g/day continuous infusion) may achieve the CSF concentrations required for treating MSSA meningitis in adult patients with normal renal function. The findings also suggest that with appropriate dose adjustments based on renal function, cefazolin can be an effective therapeutic option for MSSA meningitis, particularly in cases where traditional anti-staphylococcal agents are contraindicated or nafcillin/oxacillin is available but should be avoided because of their toxic risk.

## Figures and Tables

**Table 1:** Pharmacokinetic/pharmacodynamic breakpoints (100% T > MIC) of cefazolin in cerebrospinal fluid.

Cefazolin Regimen	PK/PD Breakpoint (100% T > MIC)
**CL_cr_ = 90 mL/min**	
2 g q.i.d. 0.5 h infusion (8 g/day)	0.25
2 g t.i.d. 4 h infusion (6 g/day)	0.25
2 g q.i.d. 4 h infusion (8 g/day)	0.5
6 g continuous infusion (6 g/day)	0.5
8 g continuous infusion (8 g/day)	1
10 g continuous infusion (10 g/day)	1
12 g continuous infusion (12 g/day)	1
**CL_cr_ = 60 mL/min**	
2 g t.i.d. 0.5 h infusion (6 g/day)	0.25
2 g q.i.d. 0.5 h infusion (8 g/day)	0.5
2 g t.i.d. 4 h infusion (6 g/day)	0.5
2 g q.i.d. 4 h infusion (8 g/day)	1
6 g continuous infusion (6 g/day)	1
8 g continuous infusion (8 g/day)	1
10 g continuous infusion (10 g/day)	2
**CL_cr_ = 30 mL/min**	
2 g b.i.d. 0.5 h infusion (4 g/day)	0.25
2 g t.i.d. 0.5 h infusion (6 g/day)	1
2 g b.i.d. 4 h infusion (4 g/day)	0.5
2 g t.i.d. 4 h infusion (6 g/day)	2
4 g continuous infusion (4 g/day)	1
6 g continuous infusion (6 g/day)	2
8 g continuous infusion (8 g/day)	2

Pharmacokinetic/pharmacodynamic breakpoints are defined as the largest MIC attaining more than 90% probability. CL_cr_; Creatinine clearance, T > MIC; time above minimum inhibitory concentration.

**Table 2:** Population pharmacokinetic parameters.

	Estimate	(RSE %)	95%CI
**Fix effects parameter**			
**CL(L/h) ^a^ = θ_CL_ × (CL_cr_/80)^θCLcr on CL^**			
*θ*_CL_(L/h)	2.86 Fixed	-	-
*θ*_CLcr on CL_	0.79 Fixed	-	-
**V_central_ (L) ^a^** = *θ*_Vc_	5.2 Fixed	-	-
**Q (L/h) ^a^** = *θ*_Q_	10.9 Fixed	-	-
**V_peripheral_ (L) ^a^** = *θ*_Vp_	4.56 Fixed	-	-
**KP_CSF_ ^b^** = *θ*_KPCSF_	0.0525 Fixed	-	-
**Q_CSF_ (L/h) ^c^** = *θ*_QCSF_	0.021 Fixed	-	-
**V_CSF_ (L) ^c^** = *θ*_VCSF_	0.092 Fixed	-	-
**Interindividual variability (exponential error model)**			
ηCL **^a^**	0.102 Fixed	-	-
ηV_central_ **^a^**	0.325 Fixed	-	-
ηQ **^a^**	0.437 Fixed	-	-
ηV_peripheral_ **^a^**	0.01 Fixed	-	-
ηKP_CSF_	1.39	(203.0)	0.0188–43.6
ηQ_CSF_ **^c^**	1.22	(0.75)	0.00245–172.7
ηV_CSF_ **^c^**	1.22	(0.38)	0.00443–178.4
**Residual variability (proportion error model)**			
ε_proportional_ **^a^**	0.0144 Fixed	-	-

^a^, parameters derived from [25]; ^b^, parameter derived from CSF/serum ratio estimated from literature data [18]; ^c^, parameter derived from [26] CI, confidence interval determined from sampling importance resampling algorithm (sampling: *n* = 1000 and resampling *n* = 1000); RSE, relative standard error; θ, population mean value; η, random variable which is normally distributed with a mean of zero and variance; ε, random error which is normally distributed with a mean of zero and variance; V_c_ and V_p_, distribution volumes of the central and peripheral compartments (L); V_CSF_, CSF volume (L); Q, central-peripheral intercompartmental clearances (L/h); Q_CSF_, CSF flow (L/h); KP_CSF_, CSF-to-plasma partition coefficient.

## Data Availability

Data supporting the findings of this study were derived from the resources available in the public domain.

## Supplementary Materials

The following supporting information can be downloaded at: https://www.mdpi.com/article/10.3390/antibiotics14101008/s1, Table S1: External PD validation of high-dose cefazolin. Table S2: Probabilities of exceeding a neurotoxicity-related cefazolin concentration (C_min_ > 64 µg/mL [20]) in CSF. The neurotoxicity-related CSF concentration value was set using the median of cefazolin CSF concentrations in three cases of generalized seizures. Table S3: Demographic information of literature data used in this study. Figure S1: Hybrid model building using $PRIOR subroutine.

## Abbreviations

The following abbreviations are used in this manuscript:

CNS	Central nervous system
CSF	Cerebrospinal fluid
CL_cr_	Creatinine clearance
PRED	Population-predicted concentration
CWRES	Conditional weighted residuals
MSSA	Methicillin-susceptible Staphylococcus aureus
PK	Pharmacokinetic
PD	Pharmacodynamic
MIC	Minimum inhibitory concentration
KP_CSF_	CSF-to-plasma partition coefficient
Q_CSF_	CSF flow
V_CSF_	CSF volume
CL	Clearance
V_central_	Volumes of distribution volumes of the central
Q	central–peripheral intercompartmental clearance
V_peripheral_	Volumes of distribution volumes of the peripheral

## References

[1] Gilbert D.N., Chambers H.F., Saag M.S., Pavia A.T., Boucher H.W. (2021). The Sanford Guide to Antimicrobial Therapy.

[2] Tunkel A.R., Hasbun R., Bhimraj A., Byers K., Kaplan S.L., Scheld W.M., van de Beek D., Bleck T.P., Garton H.J.L., Zunt J.R. (2017). 2017 Infectious Diseases Society of America’s Clinical Practice Guidelines for Healthcare-Associated Ventriculitis and Meningitis. Clin. Infect. Dis..

[3] Tunkel A.R., Hartman B.J., Kaplan S.L., Kaufman B.A., Roos K.L., Scheld W.M., Whitley R.J. (2004). Practice guidelines for the management of bacterial meningitis. Clin. Infect. Dis..

[4] (2007). Nafcillin.

[5] Li J., Echevarria K.L., Hughes D.W., Cadena J.A., Bowling J.E., Lewis J.S. (2014). Comparison of cefazolin versus oxacillin for treatment of complicated bacteremia caused by methicillin-susceptible *Staphylococcus aureus*. Antimicrob. Agents Chemother..

[6] McDanel J.S., Roghmann M.C., Perencevich E.N., Ohl M.E., Goto M., Livorsi D.J., Jones M., Albertson J.P., Nair R., O’Shea A.M.J. (2017). Comparative Effectiveness of Cefazolin Versus Nafcillin or Oxacillin for Treatment of Methicillin-Susceptible *Staphylococcus aureus* Infections Complicated by Bacteremia: A Nationwide Cohort Study. Clin. Infect. Dis..

[7] Loubet P., Burdet C., Vindrios W., Grall N., Wolff M., Yazdanpanah Y., Andremont A., Duval X., Lescure F.X. (2018). Cefazolin versus anti-staphylococcal penicillins for treatment of methicillin-susceptible *Staphylococcus aureus* bacteraemia: A narrative review. Clin. Microbiol. Infect..

[8] Shi C., Xiao Y., Zhang Q., Li Q., Wang F., Wu J., Lin N. (2018). Efficacy and safety of cefazolin versus antistaphylococcal penicillins for the treatment of methicillin-susceptible *Staphylococcus aureus* bacteremia: A systematic review and meta-analysis. BMC Infect. Dis..

[9] Weis S., Kesselmeier M., Davis J.S., Morris A.M., Lee S., Scherag A., Hagel S., Pletz M.W. (2019). Cefazolin versus anti-staphylococcal penicillins for the treatment of patients with *Staphylococcus aureus* bacteraemia. Clin. Microbiol. Infect..

[10] Thys J.P., Vanderkelen B., Klastersky J. (1976). Pharmacological study of cefazolin during intermittent and continuous infusion: A crossover investigation in humans. Antimicrob. Agents Chemother..

[11] Grégoire M., Gaborit B., Deschanvres C., Lecomte R., Deslandes G., Dailly É., Ambrosi X., Bellouard R., Asseray N., Lakhal K. (2019). High-Dosage Cefazolin Achieves Sufficient Cerebrospinal Diffusion to Treat an External Ventricular Drainage-Related *Staphylococcus aureus* Ventriculitis. Antimicrob. Agents Chemother..

[12] Le Turnier P., Gregoire M., Deslandes G., Lakhal K., Deschanvres C., Lecomte R., Talarmin J.P., Dubée V., Bellouard R., Boutoille D. (2020). NAMAP study group. Should we reconsider cefazolin for treating staphylococcal meningitis? A retrospective analysis of cefazolin and cloxacillin cerebrospinal fluid levels in patients treated for staphylococcal meningitis. Clin. Microbiol. Infect..

[13] Novak A.R., Krsak M., Kiser T.H., Neumann R.T., Cava Prado L., Molina K.C., Mueller S.W. (2021). Pharmacokinetic Evaluation of Cefazolin in the Cerebrospinal Fluid of Critically Ill Patients. Open Forum Infect. Dis..

[14] Antosz K., Battle S., Chang J., Scheetz M.H., Al-Hasan M., Bookstaver P.B. (2023). Cefazolin in the treatment of central nervous system infections: A narrative review and recommendation. Pharmacotherapy.

[15] Onita T., Ishihara N., Yano T. (2025). PK/PD-Guided Strategies for Appropriate Antibiotic Use in the Era of Antimicrobial Resistance. Antibiotics.

[16] Craig W.A. (1998). Pharmacokinetic/pharmacodynamic parameters: Rationale for antibacterial dosing of mice and men. Clin. Infect. Dis..

[17] Craig W.A. (2001). Does the dose matter?. Clin. Infect. Dis..

[18] Ikuno H., Nin K., Nishimura S. (1977). Penetration of cefazolin into cerebrospinal fluid and brain tissue. Med. Consult. New Remedies.

[19] Pitcock C., Burgess D.S., Olney K.B. (2025). Optimizing cefazolin dosing for central nervous system infections: Insights from population pharmacokinetics and Monte Carlo simulations. Antimicrob. Agents Chemother..

[20] Bechtel T.P., Slaughter R.L., Moore T.D. (1980). Seizures associated with high cerebrospinal fluid concentrations of cefazolin. Am. J. Hosp. Pharm..

[21] Ohata Y., Tomita Y., Sunakawa K., Drusano G.L., Tanigawara Y. (2019). Cerebrospinal pharmacokinetic and pharmacodynamic analysis of efficacy of meropenem in paediatric patients with bacterial meningitis. Int. J. Antimicrob. Agents.

[22] Onita T., Ikawa K., Nakamura K., Nishikawa G., Kobayashi I., Ishihara N., Tamaki H., Yano T., Naora K., Morikawa N. (2021). Prostatic Pharmacokinetic/Pharmacodynamic Evaluation of Ampicillin-Sulbactam for Bacterial Prostatitis and Preoperative Prophylaxis. J. Clin. Pharmacol..

[23] Onita T., Ikawa K., Ishihara N., Tamaki H., Yano T., Naora K., Morikawa N. (2023). Pulmonary Pharmacokinetic and Pharmacodynamic Evaluation of Ampicillin/Sulbactam Regimens for Pneumonia Caused by Various Bacteria, including Acinetobacter baumannii. Antibiotics.

[24] Onita T., Nakamura K., Nishikawa G., Ishihara N., Tamaki H., Yano T., Naora K., Morikawa N., Ikawa K. (2025). Prostatic Pharmacokinetic and Pharmacodynamic Analysis of Ceftazidime: Dosing Strategy for Bacterial Prostatitis. J. Clin. Pharmacol..

[25] Lanoiselée J., Chaux R., Hodin S., Bourayou S., Gibert A., Philippot R., Molliex S., Zufferey P.J., Delavenne X., Ollier E. (2021). Population pharmacokinetic model of cefazolin in total hip arthroplasty. Sci. Rep..

[26] Cutler R.W.P., Page L., Galicich J., Watters G.V. (1968). Formation and absorption of cerebrospinal fluid in man. Brain.

[27] Chan Kwong A.H.P., Calvier E.A.M., Fabre D., Gattacceca F., Khier S. (2020). Prior information for population pharmacokinetic and pharmacokinetic/pharmacodynamic analysis: Overview and guidance with a focus on the NONMEM PRIOR subroutine. J. Pharmacokinet. Pharmacodyn..

[28] Marshall S., Macintyre F., James I., Krams M., Jonsson N.E. (2006). Role of mechanistically-based pharmacokinetic/pharmacodynamic models in drug development: A case study of a therapeutic protein. Clin. Pharmacokinet..

[29] Lindbom L., Ribbing J., Jonsson E.N. (2004). Perl-speaks-NONMEM (PsN)—A Perl module for NONMEM related programming. Comput. Methods Programs Biomed..

[30] Four Academic Societies Joint Antimicrobial Susceptibility Surveillance Program. https://www.3ssp.jp/database/surgical/mic-5090/.

