# Supplementary material for: Cerebrospinal Pharmacokinetic Modeling and Pharmacodynamic Simulation of High-Dose Cefazolin for Meningitis Caused by Methicillin-Susceptible Staphylococcus aureus
Source: Antibiotics (Basel). 2025 Oct 11;14(10):1008. doi: 10.3390/antibiotics14101008 (PMC12561440; doi:10.3390/antibiotics14101008)
Supplement: Supplementary file 1 [file antibiotics-14-01008-s001.zip › antibiotics-3865141-supplementary.pdf]

Supplementary Table S1. External PD validation of high-dose cefazolin

Literature	・Patient information<br>・Cefazolin administration	CSF observations	CSF predicted values
**Grégoire M et al. [11]**	・a case report with normal renal function (glomerular filtration rate: 60 to 106 mL/min per 1.73 m^2 after six weeks)<br>・continuous infusion of 8 g daily	6.1 μg/mL)	Median: 5.7 μg/mL<br>(95% CI: 0.5 to 62.4 μg/mL)<br>in typical patients with CL_{cr} = 90 mL/min
**Le Turnier P et al. [12]**	・Eights subjects with normal renal function (the median glomerular filtration: 136 mL/min)<br>・continuous infusions of 8 g daily	Median: 2.8 μg/mL)<br>(ranging from 2.1 to 5.2 μg/mL)	Median: 5.7 μg/mL<br>(95% CI: 0.5 to 62.4 μg/mL)<br>in typical patients with CL_{cr} = 90 mL/min
**Novak AR et al. [13]**	・Fifteen subjects with normal renal function (median CL_{cr}: 115 mL/min)<br>・2 g three times daily (6 g/day)	Median: 1.59 μg/mL (C_{min})<br>(ranging from 0.7 to 2.2 μg/mL).	Median: 1.2 μg/mL<br>(95% CI: 0.05-25.1 μg/mL)<br>in typical patients with CL_{cr} = 90 mL/min

Supplementary Table S2. Probabilities of exceeding a neurotoxicity-related cefazolin concentration (C_{min} > 64 µg/mL [20]) in CSF. The neurotoxicity-related CSF concentration value was set using the median of cefazolin CSF concentrations in three cases of generalized seizures.

**Cefazolin regimen**	Probabilities (%) of exceeding the neurotoxicity-related CSF concentration (C_{min} > 64 µg/mL [20])
**CL_{cr} = 90 mL/min**	
**2 g q.i.d. 0.5-h infusion (8 g/day)**	1.1
**2 g t.i.d. 4-h infusion (6 g/day)**	0.8
**2 g q.i.d. 4-h infusion (8 g/day)**	1.8
**6 g continuous infusion (6 g/day)**	1.4
**8 g continuous infusion (8 g/day)**	2.5
**10 g continuous infusion (10 g/day)**	4.0
**12 g continuous infusion (12 g/day)**	5.0
**CL_{cr} = 60 mL/min**	
**2 g t.i.d. 0.5-h infusion (6 g/day)**	1.0
**2 g q.i.d. 0.5-h infusion (8 g/day)**	2.3
**2 g t.i.d. 4-h infusion (6 g/day)**	1.6
**2 g q.i.d. 4-h infusion (8 g/day)**	3.0
**6 g continuous infusion (6 g/day)**	2.6
**8 g continuous infusion (8 g/day)**	4.3
**10 g continuous infusion (10 g/day)**	6.1
**CL_{cr} =30 mL/min**	
**2 g b.i.d. 0.5-h infusion (4 g/day)**	1.1
**2 g t.i.d. 0.5-h infusion (6 g/day)**	3.7
**2 g b.i.d. 4-h infusion (4 g/day)**	1.8
**2 g t.i.d. 4-h infusion (6 g/day)**	9.0
**4 g continuous infusion (4 g/day)**	3.5
**6 g continuous infusion (6 g/day)**	6.6
**8 g continuous infusion (8 g/day)**	9.9

Supplementary Table S3 Demographic information of literature data used in this study

	PK model parameter in blood concentrations<br>・Lanoiselée J et al. [25])	Physiological parameters<br>・Cutler RWP et al. [26]	Calculation of KP_{CSF}<br>・Ikuno H et al. [18]
**Patient demographics**			
Study design	Observational study	Observational study	Observational study
The number of the subjects	N = 100 (51 males and 49 females)	N = 12 (8 males and 4 females)	N = 8 (4 males and 4 females)
Subject characteristics	Adult patients undergoing total hip arthroplasty	Eight patients with subacute sclerosing panencephalitis and four patients with pontine gliomas	adult patients with suspected meningitis.
Age (years)	67 (mean value) (range from 24 to 91)	8.3 (mean value) (range from 4 to13)	47.9 (mean value) (range from 38 to 60)
Body weight (kg)	76 (mean value) (range from 48 to 123)	24.6 (mean value) (range from 16.0 to 37.5)	NA
Renal function	Creatinine clearance (mL/min/1.73m^2): 83 (range from 17 to 129)	NA	NA
Hepatic function	NA	NA	NA
**Bioanalytical methods**	Measurement for plasma concentration: liquid chromatography system	NA	Measurement for CSF concentrations: bioassay (B. subtilis ATCC 6633)
**PK model structure**	A 2-compartment model best described cefazolin concentrations out of 1-, 2- and 3- compartment models.	NA	NA
**Covariate analysis**	Covariate for clearance in final model: Creatinine Clearance (mL/min) according to the CKD-EPI formula<br>Age, total body weight, body mass index, lean body weight, creatinine clearance (mL/min) according to the Cockcroft-Gault formula, and sex were investigated as other covariates with stepwise procedure.	NA	NA

NA; Not applicable

Supplementary Figure S1. Hybrid model building using $PRIOR subroutine

```
---------------------------------------------------------------------------------------------
$PRIOR NWPRI
$THETAP
2.86 FIXED ;(1)CL
0.79 FIXED ;(2)CCR ON CL
5.2  FIXED ;(3)V1
10.9 FIXED ;(4)Q
4.56 FIXED ;(5)V2
0.0525 FIXED ;(6)KCSF
0.021  FIXED ;(7)QCSF
0.092  FIXED ;(8)VCSF

$THETAPV ; informative
0.142
0.0902
0.186
0.360
0.288
0.0548
0.0012
0.0273

$OMEGAP  (0.102 FIXED) (0.325 FIXED) (0.437 FIXED) (0.01 FIXED)(1.387)(1.222)(1.221)
$OMEGAPD (390.5 FIXED) (254.7 FIXED) (89.8 FIXED) (3.92 FIXED) (0.486) (35285.5) (140911.2)
$SIGMAP  0.0144 FIXED
$SIGMAPD (886.4 FIXED)
---------------------------------------------------------------------------------------------
```

・**The comparison of model parameter and RSE% with prior or without prior**

with prior

Interindividual variability (exponential error model)			
	**Estimate**	**RSE (%)**	**95%CI**
ηKP_{CSF}	1.39	(18.0)	7.19-1310.6
ηQ_{CSF}^{c}	1.22	(0.75)	115.3-1133.2
ηV_{CSF}^{c}	1.22	(0.38)	134.7-925.8

without prior

Interindividual variability (exponential error model)			
	**Estimate**	**RSE (%)**	**95%CI**
ηKP_{CSF}	1.39	(203.0)	0.0188-43.6
ηQ_{CSF}^{c}	1.22	(0.75)	0.00245-172.7
ηV_{CSF}^{c}	1.22	(0.38)	0.00443-178.4

CI, confidence interval determined from sampling importance resampling algorism; RSE, relative standard error; η, random variable which is normally distributed with a mean of zero and variance
